# Mitochondrial dysfunction in liver failure requiring transplantation

**DOI:** 10.1007/s10545-016-9927-z

**Published:** 2016-04-06

**Authors:** Maria Lane, Veronika Boczonadi, Sahar Bachtari, Aurora Gomez-Duran, Thorsten Langer, Alexandra Griffiths, Stephanie Kleinle, Christine Dineiger, Angela Abicht, Elke Holinski-Feder, Ulrike Schara, Patrick Gerner, Rita Horvath

**Affiliations:** 1 Institute of Genetic Medicine, John Walton Muscular Dystrophy Research Centre and Wellcome Trust Centre for Mitochondrial Research, Central Parkway, NE1 3BZ Newcastle upon Tyne, UK; 2 Department of Paediatric Gastroenterology, University of Duisburg-Essen, Essen, Germany; 3 Department for Neuropediatrics and Muscular Diseases, Center for Pediatrics and Adolescent Medicine University Medical Center Freiburg, Freiburg, Germany; 4 Medical Genetic Center Munich, Munich, Germany; 5 Department of Paediatric Neurology, University of Duisburg-Essen, Essen, Germany; 6 Paediatric Gastroenterology/Hepatology, University of Freiburg, Freiburg, Germany

## Abstract

Liver failure is a heterogeneous condition which may be fatal and the primary cause is frequently unknown. We investigated mitochondrial oxidative phosphorylation in patients undergoing liver transplantation. We studied 45 patients who had liver transplantation due to a variety of clinical presentations. Blue native polyacrylamide gel electrophoresis with immunodetection of respiratory chain complexes I-V, biochemical activity of respiratory chain complexes II and IV and quantification of mitochondrial DNA (mtDNA) copy number were investigated in liver tissue collected from the explanted liver during transplantation. Abnormal mitochondrial function was frequently present in this cohort: ten of 40 patients (25 %) had a defect of one or more respiratory chain enzyme complexes on blue native gels, 20 patients (44 %) had low activity of complex II and/or IV and ten (22 %) had a reduced mtDNA copy number. Combined respiratory chain deficiency and reduced numbers of mitochondria were detected in all three patients with acute liver failure. Low complex IV activity in biliary atresia and complex II defects in cirrhosis were common findings. All six patients diagnosed with liver tumours showed variable alterations in mitochondrial function, probably due to the heterogeneity of the presenting tumour. In conclusion, mitochondrial dysfunction is common in severe liver failure in non-mitochondrial conditions. Therefore, in contrast to the common practice detection of respiratory chain abnormalities in liver should not restrict the inclusion of patients for liver transplantation. Furthermore, improving mitochondrial function may be targeted as part of a complex therapy approach in different forms of liver diseases.

**Electronic supplementary material:**

The online version of this article (doi:10.1007/s10545-016-9927-z) contains supplementary material, which is available to authorized users.

## Introduction

Liver failure is a rare but life-threatening critical illness requiring intensive care that occurs when large parts of the liver become severely damaged resulting in severe liver dysfunction. Symptoms include jaundice, encephalopathy, bleeding problems, fatigue and lactic acidosis. Treatment of liver failure is symptomatic, however, transplantation can be lifesaving in severe cases (Chinnery and DiMauro 2005; Fellman and Kotarsky 2011).

Although the cause of liver failure is often unknown, inherited disorders of mitochondrial oxidative phosphorylation, fatty acid oxidation, the urea cycle or glycogen storage may be responsible, especially in childhood (Casey et al 2012). Other causes of liver failure include biliary atresia, cirrhosis, tumours, intoxications and infections such as cytomegalovirus, adenovirus and hepatitis A, B and C (Iwama et al 2011).

As the liver is a vital organ with a wide range of functions it is highly dependent on ATP and a functioning oxidative phosphorylation system (Chinnery and DiMauro 2005). In general, when liver disease occurs with extra-hepatic involvement there is more reason to suspect a mitochondrial condition (Rahman 2013), however, isolated hepatic failure may also be related to mitochondrial dysfunction (Tables 1 and 2). Mitochondrial hepatopathies are most frequently caused by defects of mitochondrial DNA (mtDNA) maintenance such as mtDNA deletion (Pearson syndrome) and depletion (Rahman 2013). Genetic forms of mtDNA depletion are associated with a predominant hepatopathy, however, other organs (including muscle and brain) may also be involved (Fellman and Kotarsky 2011), such as in epileptic encephalopathy, liver failure and visual impairment in Alpers-Huttenlocher syndrome due to autosomal recessive *POLG* mutations (Naviaux and Nguyen 2004). Other defects of *POLG* are associated with valproate induced liver failure, further in support that mtDNA replication is essential for optimal hepatocyte function. Deoxyguanosine kinase (*DGUOK*) deficiency causes mtDNA depletion with a predominant liver phenotype though neurological features (nystagmus, muscular hypotonia, psychomotor retardation) often accompany this condition (Dimmock et al 2008). In patients carrying *MPV17* mutations hepatopathy is present with poor feeding, hypoglycaemia, hypotonia and faltering growth and central nervous system involvement usually appears later in the disease course (Uusimaa et al 2014). *C10orf2* and *SUCLG1* deficiency may also result in an early-onset multisystem mitochondrial hepatoencephalomyopathy with hepatic mtDNA depletion (Fellman and Kotarsky 2011; Van Hove et al 2010). Dysfunction of mitochondrial translation may also account for severe infantile liver failure (Kemp et al 2011) caused by defects in mitochondrial translation elongation factors (*GFM1*, *TSFM*) (Balasubramaniam et al 2012; Vedrenne et al 2012). A unique reversible infantile hepatopathy has been shown in association with mutations in the mitochondrial tRNA modifying factor *TRMU* (Zeharia et al 2009; Schara et al 2011). In addition, liver dysfunction has been associated with defects in mitochondrial proteins involved in single respiratory chain complexes, such as *SCO1* (complex IV assembly factor) and *BCS1L* (complex III assembly factor) (Rahman 2013). The high number of mitochondrial disease genes affecting the liver highlights the importance of mitochondria in liver cell function.

**Table 1:** Summary of mitochondrial causes of liver failure with respiratory chain deficiency

Type of mitochondrial dysfunction	Name	Genes involved	Respiratory chain defect
Disorders of mtDNA maintenance	Hepatocerebral mitochondrial disease	*DGUOK, MPV17, POLG, SUCLG1, C10ORF2*	Combined RC defect
	Pearson syndrome	Single mtDNA deletion	Combined RC defect
	Alpers-Huttenlocher syndrome	*POLG*	Combined RC defect or normal
Disorders of mitochondrial protein synthesis	Reversible infantile mitochondrial hepatopathy	*TRMU*	Combined RC defect
	Mitochondrial tRNA synthetase defects	*EARS2, FARS2*	
	Nuclear translation initiation-elongation factors	*GFM1, TSFM*	Combined RC defect
Defects of OXPHOS complex assembly	Complex III assembly	*BCS1L*	Complex III
	Complex IV assembly	*SCO1*	Complex IV

**Table 2:** Number of patients with deficiencies on BN PAGE, respiratory chain enzyme activities and mtDNA copy numbers in the cohort of 45 patients

Diagnosis (number of patients)	BN PAGE <50 %	Complex II and IV <50 %	mtDNA copy number <30 %
Acute liver failure (3)	3/3 (100 %)	3/3 (100 %)	3/3 (100 %)
Biliary atresia (9)	1/8 (13 %)	5/9 (56 %)	2/9 (22 %)
Cirrhosis (11)	3/10 (30 %)	6/11 (55 %)	2/11 (18 %)
Tumour (6)	3/5 (60 %)	2/6 (33 %)	2/6 (33 %)
Other (16)	0	4/16 (25 %)	1/16 (6 %)
Total	10/40 (25 %)	20/45 (44 %)	10/45 (22 %)

Patients with mitochondrial liver diseases usually present with defects of the respiratory chain enzymes in liver tissue. However, the role of mitochondrial dysfunction in the pathomechanism of severe liver disease of non-mitochondrial or unknown origin leading to severe liver failure has not been investigated in detail before. This study investigates mitochondrial function in liver in a large cohort of 45 patients undergoing liver transplantation due to severe liver disease of various aetiologies.

## Materials and methods

Liver samples from 45 patients undergoing liver transplantation were investigated (Supplementary Table 1). The samples were collected within 15 min after transplantation at the liver transplantation unit of the University of Essen after obtaining patient consent, and were immediately frozen and were kept at −80 °C until sample preparation. Liver samples were taken from better preserved areas, without obvious tissue destruction and routine histological examination detected end stage liver disease. Control liver samples were collected from donated liver samples of healthy individuals (usually relatives of patients). These samples were collected, stored and processed by the same methods as the patient samples. To avoid artefacts due to inappropriate storage or sample handling, all analysed samples were collected, kept frozen and analysed within a period of 12 months. The study has been approved by the local research ethics committee. We did not apply special selection criteria; we included patients undergoing liver transplantation in our transplantation unit who agreed to study participation and signed the informed consent form.

### Analysis of mtDNA copy number

Measurement of mtDNA copy number was performed on DNA extracted from liver as reported previously (Bulst et al 2009). We used 12 control liver DNA samples for determining the control range.

### Mitochondrial extraction from liver tissue and blue native preparation

Unless specified all chemicals were purchased from Sigma (Sigma Aldrich, UK). Liver tissue was weighed, thawed and cut into small pieces using a scalpel and added to a glass Elvehjem potter with ten volumes of Buffer A (0.32 M Sucrose, 10 mM Tris–HCl and 1 mM EDTA) to the weight of tissue according to the method of Fernandez-Vizarra with small modifications (Fernández-Vizarra et al 2010). Homogenisation was performed with 8 up and down strokes at 600 rpm. The homogenate was then centrifuged at 1000 g for 5 min. The supernatant was equally divided between tubes for activity assay and BN PAGE. The remaining supernatant for BN PAGE was centrifuged at 9000 g for 10 min and the pellet resuspended in 100 uL buffer A with digitonin (1 in 200 of weight of tissue). Each sample was incubated on ice for 10 min and then vortexed; 1 ml of a protease inhibitor tablet (Roche, UK) in 10 ml PBS (PI/PBS) was added to dilute the digitonin and the sample was centrifuged at 10,000 g for 10 min. The pellet was resuspended in 30–100 uL of MB2 buffer (0.5 ml 3× gel buffer (1.5 M aminocaproic acid, 150 mMBis-Tris, pH 7), 0.5 ml 2 M aminocaproic acid, 4 uL 500 mM EDTA) depending on its size. N-dodecyl *B*-D-maltoside in PI/PBS was added to a final concentration of 1 %, vortexed, incubated on ice for 15 min and then centrifuged at 16,000 g for 30 min. The supernatant was removed and saved (Leary and Sasarman 2009).

### Biochemical activity assays

The measurement was performed as previously described (Gómez-Durán et al 2011). Three cycles of freeze-thaw were performed on each sample. Each assay was performed on a Multiskan Ascent 96/384 Plate Reader (ThermoFisher Scientific, UK). To establish a normal control range, we performed the measurement on 12 control liver tissues. The activities of citrate synthase (CS) and complex II were measured by spectrophotometry as previously described (Gómez-Durán et al 2011). Complex IV activity was analysed as reduction of cytochrome *c* at 550 nm as previously described (Gómez-Durán et al 2011). All measured parameters were expressed in specific activity per mg/protein in the sample. Total protein was quantified by method of Bradford assay.

### BN PAGE and immunoblotting

Electrophoresis of proteins was carried out in 3 to 15 % gradient gels and 4 % stacking gel were prepared for BN PAGE according to the protocol of Calvaruso et al (Calvaruso et al 2008). A Gilson MiniPuls 3 gradient gel mixer (Gilson, USA) was used at a speed of 5.38 ml/min. SBG buffer (750 mmol/l aminocaproic acid, 5 % Coomassie Brilliant Blue G250 Biorad, UK) was added to the samples and 2 ug of each sample used. The electrophoresis was run on a Consort EV202 power supply at 50 V for 20 min through the stacking gel, at 100 V until the blue dye front reached halfway down the gel and at 150 V until all the blue dye had run out of the gel using blue cathode buffer (400 ml cathode buffer (15 mmol/l Bis-Tris, 50 mmol/l Tricine, pH 7), 0.08 g Coomassie Brilliant Blue G250) in the upper reservoir and anode buffer (50 mmol/l Bis-Tris, pH 7) in the lower reservoir. The gel was transferred to a PVDF membrane, destained, blocked and incubated with primary antibody to the respiratory chain complexes (complex I subunit NDUFA9 – 2 ug/ml, complex II 70 kDa subunit – 0.2 ug/ml, complex III core 1 subunit – 1 ug/ml, complex IV subunit 4–1 ug/ml, complex V ATP synthase – 0.25 ug/ml (all Abcam, UK)), a secondary rabbit anti-mouse antibody (Dako, UK) (0.5 uL per ml) and then developed with ThermoScientific Pierce ECL2 Western Blotting kit or BioRad Clarity Western ECL solution. The signal was detected with the UVP BioSpectrum 500 Imaging System and the intensity quantified using Image J software. Twelve control liver samples were used as controls. Respiratory complexes were normalised to porin and to complex II, which gave similar results in 40 patients (Supplementary data 1). Because of technically not acceptable results we had to exclude five patients from this analysis.

### Statistical analysis

SigmaPlot version 11.0 (Systat Software, UK) was used for statistical analysis. Comparisons of many groups were made using a one way ANOVA. For non-normally distributed data, a Kruskal-Wallis one way ANOVA was automatically selected. For comparisons between groups an unpaired t-test was used. A value of *p* < 0.05 was considered statistically significant (*), *p* < 0.01 very significant (**) and *p* < 0.001 extremely significant (***).

## Results

### Clinical presentation

The clinical presentations of the 45 patients, whose liver samples were analysed, are summarised in Table 2. The majority of patients were children (≤16 years) with an early onset of liver failure (30/45; 67 %). In children, biliary atresia was the most frequent diagnosis (9/30), followed by progressive familial intrahepatic cholestasis (6/30), tumour (5/30) and hyperoxaluria (4/30). The most frequent clinical presentation in the 14 adults (>16 years) was cirrhosis due to alcoholic liver disease (9/14). None of the patients showed clinical signs of a potential mitochondrial disease, and they had no increased lactate levels in serum. Patients in the acute liver disease group had isolated liver presentation and no symptoms of other organs were noted to suspect mitochondrial dysfunction. In patient 26 and 61 liver failure was triggered by drug administration, in patient 53 the primary cause could not have been identified.

### MtDNA copy number

**Fig. 1:**
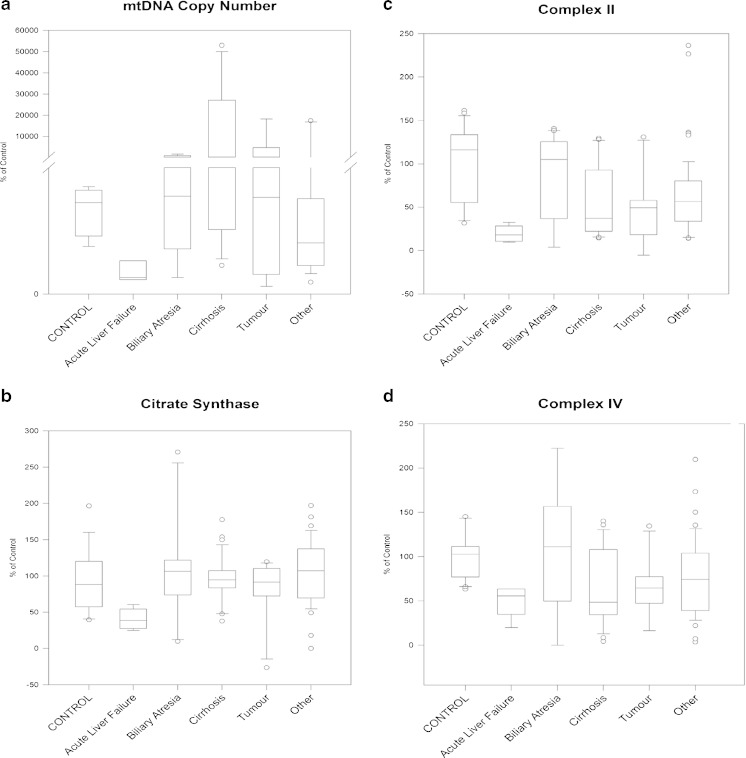
We show the distribution of mtDNA copy numbers and the activities of respiratory chain complexes II, IV and citrate synthase in 45 patients with liver failure. One way ANOVA shows the effect of liver failure on mtDNA copy number (**a**) and on the activities of complex II (**c**), complex IV (**d**) and citrate synthase (**b**). Patients are divided according to diagnosis and results are given as a percentage of the control values

MtDNA copy number showed high variability — ten samples had values ≤50 %, including all three patients with acute liver failure. In four cases with cirrhosis (2), tumour (1) or other conditions (1) we detected very high (>150 % of the control mtDNA copy numbers) (Fig. 1a). We cannot exclude that fibrosis has contributed to the low mtDNA copy numbers in some of the samples.

### Biochemical activity of respiratory chain enzymes

The activities of the respiratory chain enzymes complex II, IV and citrate synthase were measured in this study. The majority of patients studied in this cohort (40/45) had normal citrate synthase activity, an indicator of the number of functional mitochondria in the cell (Fig. 1). In contrast to citrate synthase, we measured abnormally low (<50 %) activity of complex II and/or complex IV in 20 patients (44 %), nine of them had very low (<20 %) activities (Tables 1 and 2, Fig. 1b–d).

### BN PAGE

**Fig. 2:**
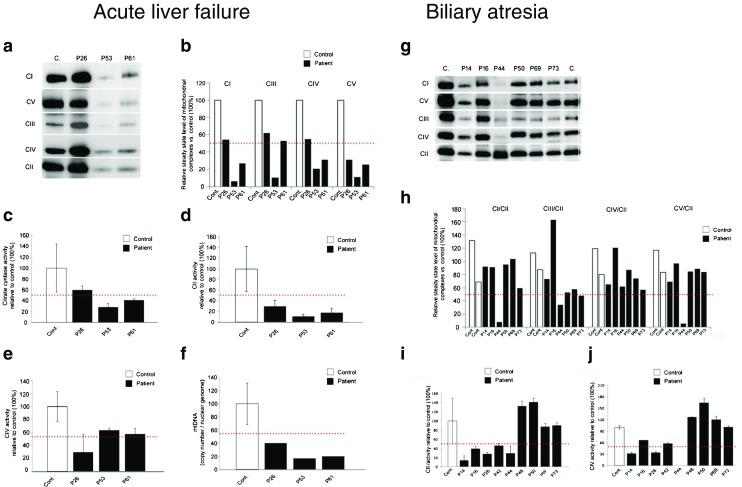
BN PAGE analysis and measurement of enzyme activities in acute liver failure in acute liver failure and biliary atresia. **Acute liver failure. a** Respiratory complexes isolated from control and patients with acute liver failure were separated using blue native gel electrophoresis on gradient Bis-Tris acrylamide gels. **b** Quantification of respiratory complexes was carried out with band densitometry. Graphs show the level of complexes relative to the control samples. Porin was used as a loading control. *Red dotted line* indicates 50 % of the control samples. Complex activities were measured spectrophotometrically as described in Methods. Data presented in percent changes relative to the control samples. **c** Citrate synthase activity. Combined data from all three patients shows a significant decrease (*p* = 0.045) in activity when compared to the control group. **d** Complex II activity. Combined data from all three patients shows a significant decrease (*p* = 0.008) in activity when compared to the control group. **e** Complex IV activity. Combined data from all three patients shows a significant decrease (*p* < 0.017) in activity when compared to the control group. **f** mt-DNA copy number. *Error bars* represent standard deviation. **Biliary atresia. g** Mitochondrial complexes isolated from control and liver failure patients’ liver were separated using blue native gel electrophoresis on gradient Bis-Tris acrylamide gels. **h** Quantification of respiratory complexes was carried out with band densitometry. Graphs represent the level of complexes relative to the control samples. Band intensities were normalised to complex II. *Red dotted line* indicates 50 % of the control samples. **i** Complex II activity. **j** Complex IV activity. *Error bars* represent standard deviation

**Fig. 3:**
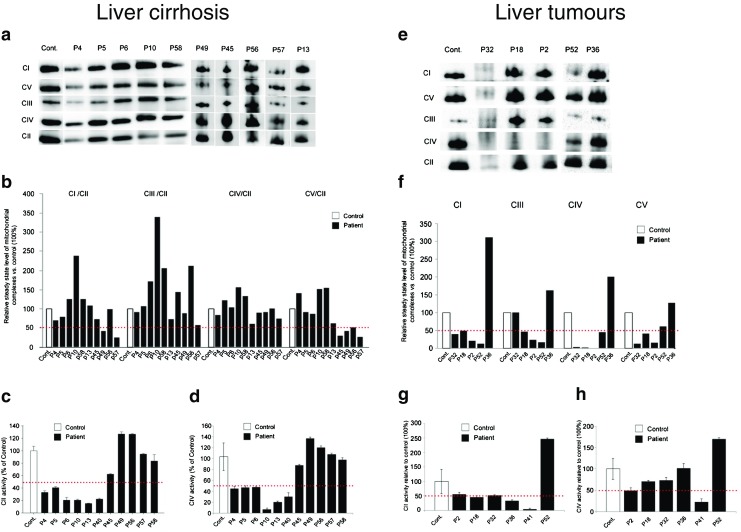
BN PAGE and measurement of enzyme activities in cirrhosis and liver tumours. **Liver cirrhosis. a** Mitochondrial complexes isolated from control and cirrhosis patients’ liver were separated using *blue* native gel electrophoresis on gradient Bis-Tris acrylamide gels. **b** Quantification of respiratory complexes was carried out with band densitometry. Graphs represent the level of complexes relative to the control samples. Band intensities were normalised to complex II. *Red dotted line* indicates 50 % of the control samples. **c** Complex II activity. **d** Complex IV activity. *Error bars* represent standard deviation. L**iver tumours. e** Mitochondrial complexes isolated from control and tumour patients’ liver were separated using *blue* native gel electrophoresis on gradient Bis-Tris acrylamide gels. **f** Quantification of respiratory complexes was carried out with band densitometry. Graphs represent the level of complexes relative to the control samples (100 %). Band intensities were normalised to complex II. *Red dotted line* indicates 50 % of the control samples. **g** Complex II activity. **h** Complex IV activity. *Error bars* represent standard deviation

In this cohort ten of 40 patients (25 %) showed mitochondrial abnormalities on BN PAGE (Tables 1 and 2, Figs. 2 and 3). A combined deficiency of many respiratory chain enzymes was the most common type, however in a few cases the decreased activity was only observed in a single enzyme affecting complexes I-IV (Figs. 2 and 3). An isolated complex V defect was not seen in any patient. Although abnormal respiratory complexes were detected in some patients in each disease category severe decreases of multiple enzymes were detected in all three patients with acute liver failure.

### Mitochondrial dysfunction in the different clinical groups

#### Acute liver failure

Two of three patients (patients 53 and 61) showed a severe decrease and one (patient 26) a milder decrease in the levels of all complexes of the respiratory chain on BN PAGE (Fig. 2a and b). In support of these data we detected significantly reduced activities of complex II (*p* = 0.008) and complex IV (*p* = 0.017) in all three patients and the activity of citrate synthase and the mtDNA copy number were also lower than controls indicating that a low number of mitochondria are present in these patients (Fig. 2c–f). In fact, mtDNA copy numbers were within the range suggestive of mtDNA depletion. Sequencing of the *DGUOK, POLG* and *MPV17* genes did not reveal the causative mutation, however, it is possible that the mtDNA depletion contributed to the liver failure in these patients.

#### Biliary atresia

In biliary atresia one of eight patients (patient 44) had a clear deficiency of complexes I and V on BN PAGE (Fig. 2g and h), which was associated with unmeasurable activity of respiratory chain complex IV (Fig. 2j). On biochemical measurement of enzyme activities two additional patients (patients 14 and 28) had a decreased complex IV activity (<50 % of control) despite normal complex IV on BN PAGE in patient 14 (Fig. 2j). The mtDNA copy number was significantly increased in patients 44 and 48, suggesting a compensatory mitochondrial proliferation. Reduced mtDNA copy number (<30 % of normal range) was found in two additional patients in this group (patients 42 and 69).

#### Cirrhosis

Three of ten patients (patients 45, 49 and 57) appeared to have <50 % levels of oxidative phosphorylation complexes on BN PAGE (Fig. 3a and b). Interestingly, the activity of complex II was significantly decreased <50 % of normal (18–40 %) in six patients with cirrhosis (Fig. 3c), while complex IV activity also indicated lower levels of activities in the same six patients (Fig. 3d). Citrate synthase activity was within the normal range in the majority of these patients, indicating that low complex II and complex IV activity is not caused by a low number of mitochondria. MtDNA copy number was also within the normal range, or even higher in most patients with cirrhosis, except for two patients, who had low mtDNA copy number in a pathological range (10–20 %) (Tables 1 and 2, Fig. 1a).

#### Tumours

In patients with tumours, results from BN PAGE were variable, likely due to the heterogeneity of tumour types in this cohort encompassing hepatocellular carcinoma (*n* = 1), hepatoblastoma (*n* = 3) and two not well characterised tumours (*n* = 2) (Fig. 3e and f). Three out of five patients had deficiencies of multiple respiratory chain enzymes on BN PAGE, and one (patient 36) had increased levels of the respiratory chain complexes I, III, IV and V.

In three of these cases (patients 36, 41 and 52) tumour tissue and non-affected healthy or cirrhotic tissue were taken from the same patient at the time of transplantation. In one of these patients (patient 52) tumour tissue showed lower expression of respiratory chain complexes on BN PAGE both compared to healthy tissue from the same liver and to controls, and in patient 36 expression of complexes I and IV was increased in tumour tissue compared to healthy tissue as well as controls confirming that the respiratory chain alterations are characteristic for the tumour cells.

Biochemical measurement of enzyme activities detected lower complexes II and/or IV activities in two out of six patients (patients 2 and 41, 33 %) with tumours, which was significant for the complex II decrease (Fig. 3g). In two patients the mtDNA copy number was decreased ≤30 %. On the contrary, patient 41 had a substantially elevated mtDNA copy number, while in the same patient biochemical measurement of complex II was significantly reduced (Fig. 3h, Tables 1 and 2, Fig. 1). This may be specific for the tumour cells in this patient.

#### Other conditions

We studied six patients with progressive familial intrahepatic cholestasis, four patients with hyperoxaluria, three patients with cystic liver and single cases with Budd Chiari syndrome, Alagille syndrome and congenital liver fibrosis. BN PAGE did not reveal significant defects in any of these patients, however we detected some decrease in complex II and/or IV activities in four patients from various diseases within the “other” group. Low mtDNA copy number (<30 %) was detected in only one patient from this group (Tables 1 and 2).

## Discussion

End stage liver disease can be caused by a variety of conditions including viral infections, inherited metabolic diseases, drug toxicity, defects of biliary ducts, cholestasis or tumours, and in many cases the aetiology remains unclear. Even in conditions with known causes such as tumours and progressive familial intrahepatic cholestasis the reasons for progression to end stage liver disease involve various pathomechanisms. We studied liver tissue samples in a large, representative cohort of 45 patients undergoing liver transplantation for various forms of liver disease and detected mitochondrial abnormalities (mitochondrial respiratory chain deficiency and/or abnormal mtDNA copy number) in several cases. This cohort is representative of a wide range of severe liver conditions and is the first to systematically investigate mitochondrial function in severely affected liver at the time of transplantation. All patients had a severe, isolated liver manifestation and no involvements of other organs supporting mitochondrial disease such as brain and skeletal muscle were noted at the time of transplantation.

In acute liver failure all three patients had abnormal mitochondrial function supported by reduced complexes on BN PAGE, decreased activities of complexes II and IV and low mtDNA copy numbers. These results indicate that mtDNA depletion may lead to mitochondrial dysfunction and acute liver cell death in these patients. Although screening for known genetic causes of hepatic mtDNA depletion did not reveal the primary molecular defect in our patients, it is still possible that mutations in novel genes or in known genes where a different clinical presentation is expected are underlying mtDNA depletion in these cases. However we cannot exclude that the detected mitochondrial abnormalities in these patients are due to a low number of mitochondria in the diseased liver cells as a result of a sudden unknown disease mechanism.

Helbling et al 2013 studied mtDNA copy numbers in 244 patients with various forms of liver disease leading to hepatic failure requiring liver transplantation. They detected low mtDNA copy numbers in 66 % of the cases and in half of these patients the mtDNA copy number was in the range of definite mtDNA depletion (Helbling et al 2013). Screening for mutations in known genes associated with mtDNA depletion revealed heterozygous variants in *POLG* and *DGUOK*, however, a causative effect of these variants to cause mtDNA depletion and liver failure has not been shown, and no other mitochondrial studies were performed in support of a mitochondrial aetiology of liver dysfunction. The authors suggest that patients with acute liver failure in association with mtDNA depletion may have an underlying genetic predisposition or a mitochondrial disease, however, no experimental evidence has been shown in support of this hypothesis.

MtDNA depletion has been detected in 50 % (50/100) of children with multiple respiratory chain enzyme deficiency and most of these patients (32/50; 64 %) presented with severe neonatal onset liver involvement (Sarzi et al 2007). However, the causative mutations could not be identified in half of these cases, illustrating further genetic heterogeneity (Sarzi et al 2007). Another study performed whole exome sequencing in three children with acute liver failure and identified pathogenic mutations in *MPV17, SERAC1* and *NOTCH2*, despite the lack of characteristic clinical phenotypes for these genes (Vilarinho et al 2014). Based on our data and previous reports rare genetic causes may be responsible for acute liver failure in a number of patients and next generation sequencing studies may define further novel mitochondrial phenotypes.

The detection of mitochondrial abnormalities in a relatively large number of patients with a wide range of diagnoses raises the possibility that oxidative phosphorylation may be a secondary result of a more complex cellular phenotype and can contribute to the hepatocellular dysfunction in various conditions. In biliary atresia we detected variable mitochondrial alterations (low complex IV activity, decreased complex I and V on BN PAGE, low mtDNA copy number) in 20–50 % of patients. However the lack of consequent findings in this disease group did not allow a better understanding of their role in the pathomechanism of the disease. It is possible that they are linked with certain stages of hepatocellular dysfunction, as supported by the detection of low mtDNA copy numbers in leukocytes of patients with early stage biliary atresia, suggesting a role of inflammatory reaction and secondary mitochondrial DNA damage in this condition (Tiao et al 2007).

Variable findings were detected in our study in patients with cirrhosis. About half of cirrhosis patients did not show any abnormalities, however, others had defects in one or more complexes or showed copy number abnormalities. The decreased activity of complex II was quite common in cirrhosis (55 %). It may be related to the disease progression, however, currently we have no explanation why complex II is most affected in this disease group. Chronic ethanol consumption has been shown to affect mitochondrial function by altering the mitochondrial permeability transition pore in the liver, suggesting a potential mechanism (King et al 2014).

In support of the secondary aetiology of mitochondrial alterations in cirrhosis, mitochondrial DNA re-arrangements and low copy numbers have been previously detected in patients with alcohol-induced end stage liver disease and their role has been suggested in the pathophysiology of the disease (Tang et al 2012).

We detected very diverse findings in liver tumours. While five out of six patients with tumours had deficient respiratory chain enzymes, one patient showed increased complexes on BN PAGE. Contrasting results were detected in the different tumours in the activity assays and two patients had lower and one patient very high mtDNA copy numbers on quantitative PCR analysis compared to the healthy tissue from the same liver, suggesting that cancer cells have altered mitochondrial metabolism. The role of mitochondria has been intensively studied in cancer and the pharmacological inhibition of mitochondrial metabolism is emerging as a potential therapeutic strategy in some cancers (Ahn and Metallo 2015). In support of our findings, somatic mtDNA mutations and decreased mtDNA copy number have been detected in hepatocellular carcinoma, suggesting that a mitochondrial dysfunction-activated signalling cascade may play an important role in the disease progression (Hsu et al 2013). A possible link between mtDNA depletion and tumorigenesis has been suggested by the detection of hepatocellular carcinoma in a patient with *DGUOK* deficiency (Freisinger et al 2006).

Our data highlight that mitochondrial dysfunction may be secondary in a wide range of liver diseases of non-mitochondrial aetiology, although exclusion of primary mitochondrial causes has only been performed in a few selected cases. Detection of respiratory chain dysfunction in liver disease requiring transplantation is not sufficient to make the diagnosis of a mitochondrial disease, and should not restrict the inclusion of patients for liver transplantation. Furthermore, recent data suggest that liver transplantation may provide clinical benefit for patients with primary mitochondrial disease, especially when the clinical presentation is likely to be restricted to liver (Dimmock et al 2008; Hynynen et al 2014).

In conclusion, this study provides good evidence that mitochondrial dysfunction is present in patients undergoing transplantation due to various types of primary liver disease. We suggest that mitochondrial disease should be investigated in patients with acute liver failure of unknown cause, although finding the molecular cause can be difficult due to genetic heterogeneity. Although mitochondrial respiratory chain deficiencies and mtDNA copy number abnormalities are helpful to identify patients with a potentially primary mitochondrial liver disease, a critical interpretation of these data is needed. Further investigation of the role of mitochondrial dysfunction in end stage liver disease in other, non-mitochondrial hepatic disorders may reveal novel pathways, which may be targeted to improve mitochondrial function and to prevent or ameliorate disease progression.

## Electronic supplementary material

Below is the link to the electronic supplementary material.

Supplementary Table 1 Summary of the clinical presentations of patients (DOCX 17.6 kb)

Supplementary Fig. 1 Immunoblotting for porin we used as loading controls for the SDS-PAGE and BN-PAGE (JPG 423 kb)

## Abbreviations

ATP: Adenosine triphosphate
mtDNA: Mitochondrial deoxyribonucleic acid
POLG: Polymerase gamma
tRNA: Transfer ribonucleic acid
EDTA: Ethylenediaminetetraacetic acid
BN PAGE: Blue native polyacrylamide gel electrophoresis
PBS: Phosphate buffered saline
MB2: Menezo’s B2 medium
DTNB: 5,5′-dithiobis-(2-nitrobenzoic acid)
DCPIP: 2,6-dichlorophenol-indophenol
SBG: Serva Blue G
PVDF: Polyvinylidene fluoride
ANOVA: Analysis of variance
PCR: Polymerase chain reaction

## References

[CR1] Ahn CS, Metallo CM (2015). Mitochondria as biosynthetic factories for cancer proliferation. Cancer Metab.

[CR2] Balasubramaniam S, Choy YS, Talib A, Norsiah MD, van den Heuvel LP, Rodenburg RJ (2012). Infantile progressive hepatoencephalomyopathy with combined OXPHOS deficiency due to mutations in the mitochondrial translation elongation factor gene GFM1. JIMD Rep.

[CR3] Bulst S, Abicht A, Holinski-Feder E, Muller-Ziermann S, Koehler U, Thirion C (2009). In vitro supplementation with dAMP/dGMP leads to partial restoration of mtDNA levels in mitochondrial depletion syndromes. Hum Mol Genet.

[CR4] Calvaruso MA, Smeitink J, Nijtmans L (2008). Electrophoresis techniques to investigate defects in oxidative phosphorylation. Methods.

[CR5] Casey JP, McGettigan P, Lynam-Lennon N, McDermott M, Regan R, Conroy J (2012). Identification of a mutation in LARS as a novel cause of infantile hepatopathy. Mol Genet Metab.

[CR6] Chinnery PF, DiMauro S (2005). Mitochondrial hepatopathies. J Hepatol.

[CR7] Dimmock DP, Dunn JK, Feigenbaum A, Rupar A, Horvath R, Freisinger P (2008). Abnormal neurological features predict poor survival and should preclude liver transplantation in patients with deoxyguanosine kinase deficiency. Liver Transpl.

[CR8] Fellman V, Kotarsky H (2011). Mitochondrial hepatopathies in the newborn period. Semin Fetal Neonatal Med.

[CR9] Fernández-Vizarra E, Ferrín G, Pérez-Martos A, Fernández-Silva P, Zeviani M, Enríquez JA (2010). Isolation of mitochondria for biogenetical studies: an update. Mitochondrion.

[CR10] Freisinger P, Fütterer N, Lankes E, Gempel K, Berger TM, Spalinger J (2006). Hepatocerebral mitochondrial DNA depletion syndrome caused by deoxyguanosine kinase (DGUOK) mutations. Arch Neurol.

[CR11] Gómez-Durán A, Pacheu-Grau D, López-Pérez MJ, Montoya J, Ruiz-Pesini E (2011). Mitochondrial pharma-Q-genomics: targeting the OXPHOS cytochrome b. Drug Discov Today.

[CR12] Helbling D, Buchaklian A, Wang J, Wong LJ, Dimmock D (2013). Reduced mitochondrial DNA content and heterozygous nuclear gene mutations in patients with acute liver failure. J Pediatr Gastroenterol Nutr.

[CR13] Hsu CC, Lee HC, Wei YH (2013). Mitochondrial DNA alterations and mitochondrial dysfunction in the progression of hepatocellular carcinoma. World J Gastroenterol.

[CR14] Hynynen J, Komulainen T, Tukiainen E, Nordin A, Arola J, Kälviäinen R (2014). Acute liver failure after valproate exposure in patients with POLG1 mutations and the prognosis after liver transplantation. Liver Transpl.

[CR15] Iwama I, Baba Y, Kagimoto S, Kishimoto H, Kasahara M, Murayama K, Shimizu K (2011). Case report of a successful liver transplantation for acute liver failure due to mitochondrial respiratory chain complex III deficiency. Transplant Proc.

[CR16] Kemp JP, Smith PM, Pyle A, Neeve VC, Tuppen HA, Schara U (2011). Nuclear factors involved in mitochondrial translation cause a subgroup of combined respiratory chain deficiency. Brain.

[CR17] King AL, Swain TM, Mao Z, Udoh US, Oliva CR, Betancourt AM (2014). Involvement of the mitochondrial permeability transition pore in chronic ethanol-mediated liver injury in mice. Am J Physiol Gastrointest Liver Physiol.

[CR18] Leary SC, Sasarman F (2009). Oxidative phosphorylation: synthesis of mitochondrially encoded proteins and assembly of individual structural subunits into functional holoenzyme complexes. Methods Mol Biol.

[CR19] Naviaux RK, Nguyen KV (2004). POLG mutations associated with Alpers’ syndrome and mitochondrial DNA depletion. Ann Neurol.

[CR20] Rahman S (2013). Gastrointestinal and hepatic manifestations of mitochondrial disorders. J Inherit Metab Dis.

[CR21] Sarzi E, Bourdon A, Chrétien D, Zarhrate M, Corcos J, Slama A (2007). Mitochondrial DNA depletion is a prevalent cause of multiple respiratory chain deficiency in childhood. J Pediatr.

[CR22] Schara U, von Kleist-Retzow JC, Lainka E (2011). Acute liver failure with subsequent cirrhosis as the primary manifestation of TRMU mutations. J Inherit Metab Dis.

[CR23] Tang C, Liang X, Liu H, Guo L, Pi R, Yang J (2012). Changes in mitochondrial DNA and its encoded products in alcoholic cirrhosis. Int J Clin Exp Med.

[CR24] Tiao MM, Lin TK, Kuo FY, Huang CC, Du YY, Chen CL, Chuang JH (2007). Early stage of biliary atresia is associated with significant changes in 8-hydroxydeoxyguanosine and mitochondrial copy number. J Pediatr Gastroenterol Nutr.

[CR25] Uusimaa J, Evans J, Smith C, Butterworth A, Craig K, Ashley N (2014). Clinical, biochemical, cellular and molecular characterization of mitochondrial DNA depletion syndrome due to novel mutations in the MPV17 gene. Eur J Hum Genet.

[CR26] Van Hove JL, Saenz MS, Thomas JA, Gallagher RC, Lovell MA, Fenton LZ (2010). Succinyl-CoA ligase deficiency: a mitochondrial hepatoencephalomyopathy. Pediatr Res.

[CR27] Vedrenne V, Galmiche L, Chretien D, de Lonlay P, Munnich A, Rotig A (2012). Mutation in the mitochondrial translation elongation factor EFTs results in severe infantile liver failure. J Hepatol.

[CR28] Vilarinho S, Choi M, Jain D, Malhotra A, Kulkarni S, Pashankar D (2014). Individual exome analysis in diagnosis and management of pediatric liver failure of indeterminate etiology. J Hepatol.

[CR29] Zeharia A, Shaag A, Pappo O, Mager-Heckel AM, Saada A, Beinat M (2009). Acute infantile liver failure due to mutations in the TRMU gene. Am J Hum Genet.

