# Supplementary material for: Mitochondrial dysfunction in liver failure requiring transplantation
Source: J Inherit Metab Dis. 2016 Apr 6;39(3):427–36. doi: 10.1007/s10545-016-9927-z (PMC4851707; doi:10.1007/s10545-016-9927-z)
Supplement: Supplementary file 1 — Summary of the clinical presentations of patients (DOCX 17.6 kb) [file 10545_2016_9927_MOESM2_ESM.docx]

Supplementary Table. Summary of the clinical presentations of patients.

| **Patient Number** | **Age** | **Diagnosis** | **Patient Number** | **Age** | **Diagnosis** |  |
| --- | --- | --- | --- | --- | --- | --- |
| **P1** | 52y | Cystic liver | **P40** | 16y | Cirrhosis |  |
| **P2** | 57y | Tumour (hepatocellular carcinoma) | **P41** | 16y | Tumour |  |
| **P3** | 43y | Budd-Chiari syndrome, Re-LT | **P42** | 11m | Biliary atresia |  |
| **P4** | 47y | Cirrhosis | **P43** | 14y | Progressive familial intrahepatic cholestasis |  |
| **P5** | 61y | Cirrhosis | **P44** | 2m | Biliary atresia |  |
| **P6** | 29y | Cirrhosis | **P45** | 49y | Cirrhosis |  |
| **P10** | 60y | Cirrhosis | **P48** | 19m | Biliary atresia |  |
| **P11** | 41y | Cystic liver | **P49** | 60y | Cirrhosis |  |
| **P13** | 6y | Cirrhosis | **P50** | 18m | Biliary atresia |  |
| **P14** | 5m | Biliary atresia | **P52** | 20m | Tumour |  |
| **P16** | 8m | Biliary atresia | **P53** | 11m | Acute liver failure |  |
| **P18** | 17m | Tumour (hepatoblastoma) | **P54** | 17m | Progressive familial intrahepatic cholestasis |  |
| **P20** | 2y | Progressive familial intrahepatic cholestasis | **P56** | 57y | Cirrhosis |  |
| **P21** | 21m | Progressive familial intrahepatic cholestasis | **P57** | 42y | Cirrhosis |  |
| **P23** | - | Hyperoxaluria | **P58** | 42y | Cirrhosis |  |
| **P24** | 6m | Hyperoxaluria | **P61** | 46y | Acute liver failure |  |
| **P25** | 3m | Hyperoxaluria | **P65** | 14y | Polycystic kidney/liver |  |
| **P26** | 6y | Acute liver failure | **P66** | 14m | Progressive familial intrahepatic cholestasis |  |
| **P28** | 7m | Biliary atresia | **P67** | 14m | Progressive familial intrahepatic cholestasis |  |
| **P32** | 11m | Tumour (hepatoblastoma) | **P69** | 3y | Biliary atresia |  |
| **P34** | 8m | Alagille syndrome | **P71** | 2y | Hyperoxaluria |  |
| **P36** | 11m | Tumour (hepatoblastoma) | **P73** | 6m | Biliary atresia |  |
| **P38/24** | 14y | Congenital liver fibrosis |  |  |  |  |
| **Summary of diagnoses** | | | **Patients (n)** | | **Age range** | |
| Acute liver failure | | | 3 | | 11m-46y | |
| Biliary atresia | | | 9 | | 2m-3y | |
| Cirrhosis | | | 11 | | 6y-61y | |
| Tumour | | | 6 | | 11m-57y | |
| Progressive familial intrahepatic cholestasis | | | 6 | | 14m-14y | |
| Hyperoxaluria | | | 4 | | 3m-2y | |
| Other | | | 6 | | 8m-52y | |
| **TOTAL** | | | **45** | | **2m-61y** | |
