# Supplementary figures and images for: Mitochondrial dysfunction in liver failure requiring transplantation
Source: J Inherit Metab Dis. 2016 Apr 6;39(3):427–36. doi: 10.1007/s10545-016-9927-z (PMC4851707; doi:10.1007/s10545-016-9927-z)

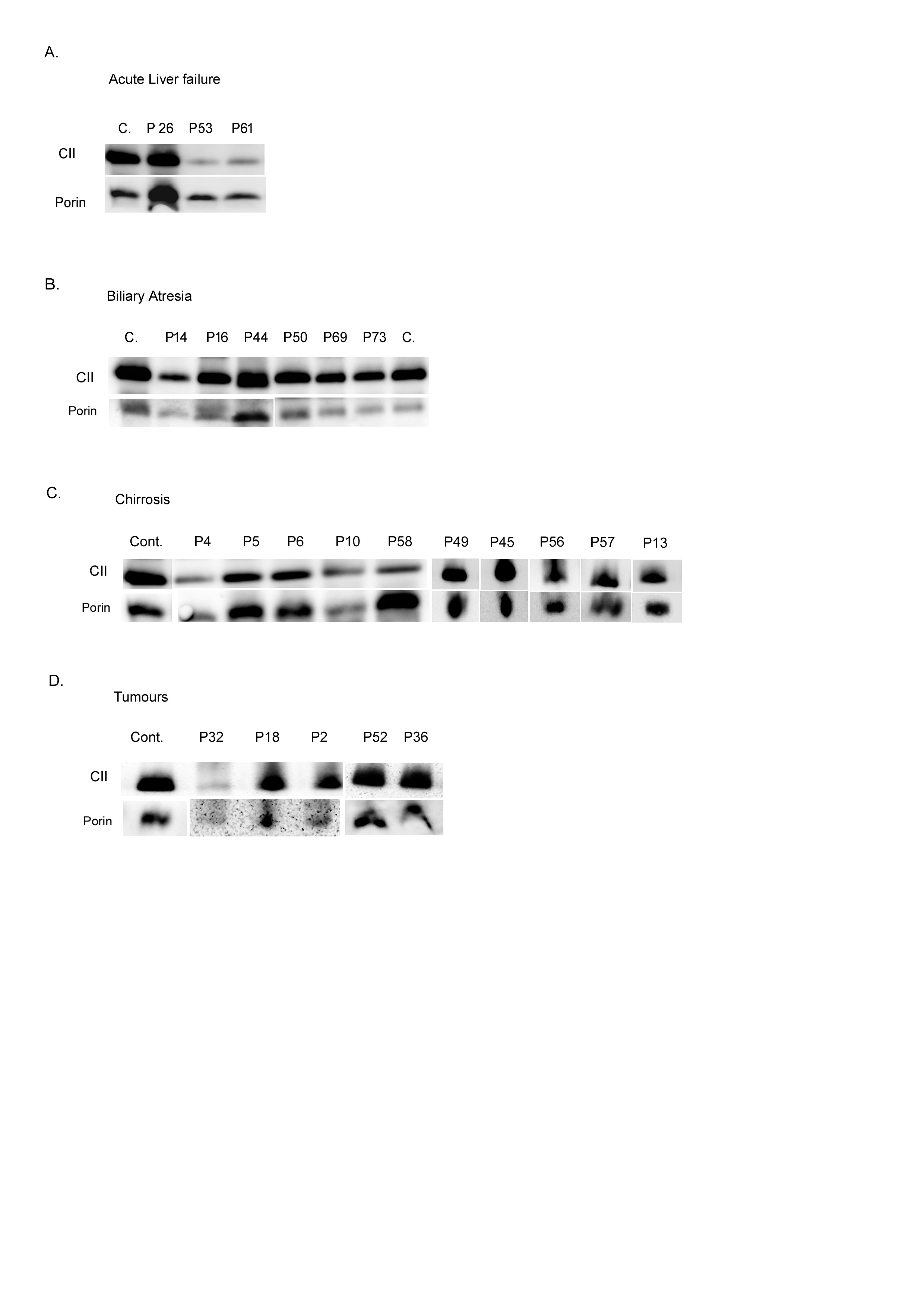

Supplement: Supplementary file 2 — Immunoblotting for porin we used as loading controls for the SDS-PAGE and BN-PAGE (JPG 423 kb) [file 10545_2016_9927_MOESM1_ESM.jpg]
